# A new t(9;11;20;22)(q34;p11.2;q11.21;q11) in a Philadelphia-positive chronic myeloid leukemia case

**DOI:** 10.3892/ol.2012.1039

**Published:** 2012-11-21

**Authors:** WALID AL-ACHKAR, ABDULSAMAD WAFA, THOMAS LIEHR

**Affiliations:** 1 Department of Molecular Biology and Biotechnology, Human Genetics Division, Atomic Energy Commission, Damascus, Syria;; 2 Jena University Hospital, Institute of Human Genetics, Jena, Germany

**Keywords:** chronic myeloid leukemia, KAI1, KIF3B, variant Philadelphia chromosome, fluorescence *in situ* hybridization, high-resolution array-proven multicolor banding, imatinib mesylate

## Abstract

The so-called Philadelphia (Ph) chromosome is found in over 90% of cases of chronic myeloid leukemia (CML). Of these cases, 2–10% demonstrate complex translocations involving a third chromosome in addition to chromosomes 9 and 22. Since the majority of CML cases are currently treated with imatinib, variant rearrangements tend to have no specific prognostic significance, although the mechanisms involved in resistance to therapy have yet to be investigated. This study evaluated a CML case with complex chromosomal aberrations not previously observed. A four-chromosome translocation involving chromosomal regions including 11p11.2 and 20q11.21 in addition to 9q34 and 22q11 was characterized in detail using array-proven multicolor banding (aMCB), a technique which has proven to be of significance in characterizing breakpoint regions in detail. Underlying mechanisms and prognostic factors are discussed.

## Introduction

Chronic myeloid leukemia (CML) is a clonal malignant disorder of a pluripotent hematopoietic stem cells and is characterized by the presence of the Philadelphia chromosome (Ph) in over 90% of cases (1). Ph is a product of reciprocal translocation between the long arms of chromosomes 9 and 22. This rearrangement combines the ABL1 proto-oncogene on chromosome 9 with the breakpoint cluster region (BCR gene) on chromosome 22. However, variant complex chromosomal translocations involving one or more chromosomes in addition to 9 and 22 are detected in 2–10% of CML cases. It is generally accepted that the clinical, prognostic and hematological features of CML with variant translocations are not distinct from those with the typical t(9;22) translocation (1).

The development of new fluorescence *in situ* hybridization (FISH) techniques has led to the identification of unexpected deletions adjacent to the translocation breakpoint on the derivative chromosome 9 [der(9)] in 10–15% of CML patients with classic Ph-positive status, and in as many as 30–40% of patients with variant Ph translocations (2). These deletions are thought to occur simultaneously as the Ph translocation rather than as a secondary event, and may involve the loss of sequences from chromosome 9, chromosome 22 or both (3). However, the deletions on der(9) are associated with a shorter duration of the chronic phase and a poor response to interferon and imatinib mesylate (4–5).

Imatinib mesylate (Glivec, formerly known as STI571) was specifically designed to inhibit the tyrosine kinase activity of the BCR/ABL protein and other tyrosine kinases, including cABL, c-KIT and platelet-derived growth factor receptor (PDGFR). Glivec inactivates downstream signaling by binding to an active site of the tyrosine kinase, halting cell proliferation and inducing apoptosis (6). Imatinib therapy has demonstrated high efficacy, achieving a complete or major cytogenetic response, i.e., a reduction to 0–34% Ph-positive cells. This positive effect was observed in cases with a simple t(9;22) translocation combined with complex translocations resulting in BCR/ABL gene fusion, as well as in cases with clonal evolution (7–8).

In this study, we report a novel Ph chromosome-positive CML case with an absence of the BCR/ABL fusion gene on der(9) and a new complex rearrangement formed by chromosomes 11 and 20 as well as 9 and 22. This unusual translocation has been characterized by FISH and array-proven multicolor banding (aMCB), the latter being extremely useful in characterizing breakpoint regions in detail. Underlying mechanisms and prognostic factors are discussed.

## Materials and methods

### Case report

A 55-year-old female was diagnosed as suffering from CML in the chronic phase in September 2007. The patient had a white blood cell count (WBC) of 3.190×10^9^/l with 46.7% neutrophils, 48.6% lymphocytes, 1.3% eosinophiles and 3.4% basophiles. The platelet count was 398×10^9^/l and the hemoglobin level was 10.3 g/dl. The patient was treated with imatinib mesylate at a dose of 400 mg/day for a period of 16 months. During that period the patient showed no symptoms. Later the patient was lost during follow-up.

### Cytogenetic analysis

Chromosome analysis using GTG-banding was performed according to standard procedures (9). A total of 20 metaphases derived from the unstimulated bone marrow of the patient were analyzed. Karyotypes were described according to the international system for human cytogenetic nomenclature (10).

### Molecular cytogenetics

FISH was performed using an LSI BCR/ABL dual-color dual-fusion translocation probe (Abbott Molecular/Vysis, Abbott Park, IL, USA) and a whole chromosome painting (WCP) probe for chromosomes 9, 11, 20 and 22 (MetaSystems, Altlussheim, Germany), was applied according to the manufacturer’s instructions (11). aMCB sets based on microdissection-derived region-specific libraries for chromosomes 9, 11, 20 and 22 were applied as previously described (12–13). A total of 20 metaphase spreads were analyzed using a fluorescence microscope (Axio Imager.Z1 mot, Zeiss, Germany) equipped with appropriate filter sets to distinguish between a maximum of five fluorochromes and the counterstain DAPI (4′,6-diamino-2-phenylindole). Image capturing and processing were carried out using an ISIS imaging system (MetaSystems) for the MCB evaluation.

### Reverse transcription-polymerase chain reaction (RT-PCR) for BCR/ABL fusion transcripts

RT-PCR was carried out as previously described (14).

## Results

**Figure 1.**
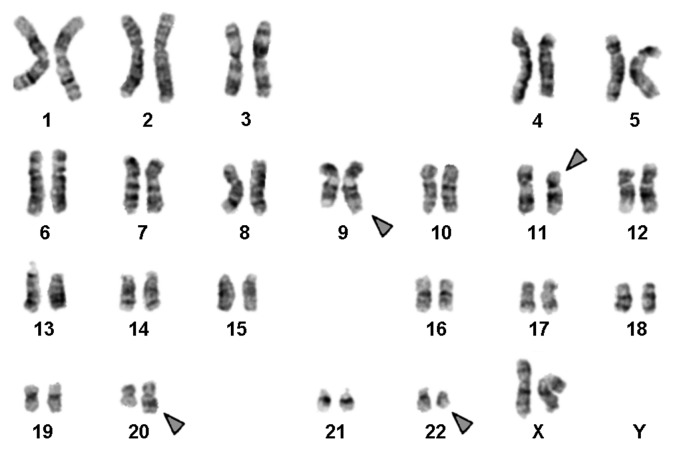
GTG-banding revealed a complex karyotype involving two chromosomes besides chromosomes 9 and 22. Derivative chromosomes are indicated by the arrow heads.

**Figure 2.**
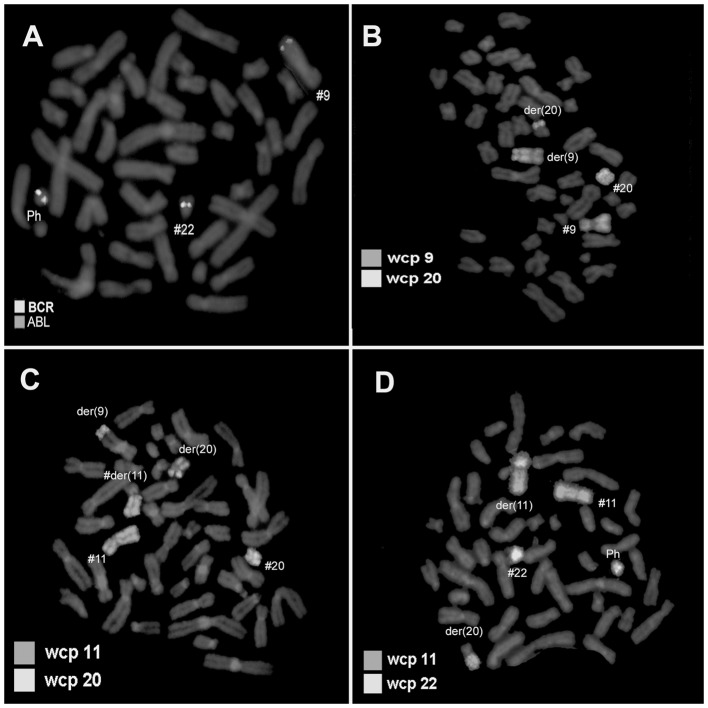
Karyotype and chromosomal aberrations were confirmed using molecular cytogenetic approaches. (A) Fluorescence *in situ* hybridization (FISH) using LSI BCR/ABL dual-color dual-fusion translocation probes, BCR (green) and ABL (red), confirmed the presence of the BCR/ABL translocation and the Philadelphia (Ph) chromosome, while the ABL/BCR fusion gene on der(9) was not observed. (B–D) The results of FISH analysis using whole chromosome painting probe sets for chromosomes 9, 11, 20 and 22. #, chromosome; der, derivative chromosome; Ph, Philadelphia chromosome.

**Figure 3.**
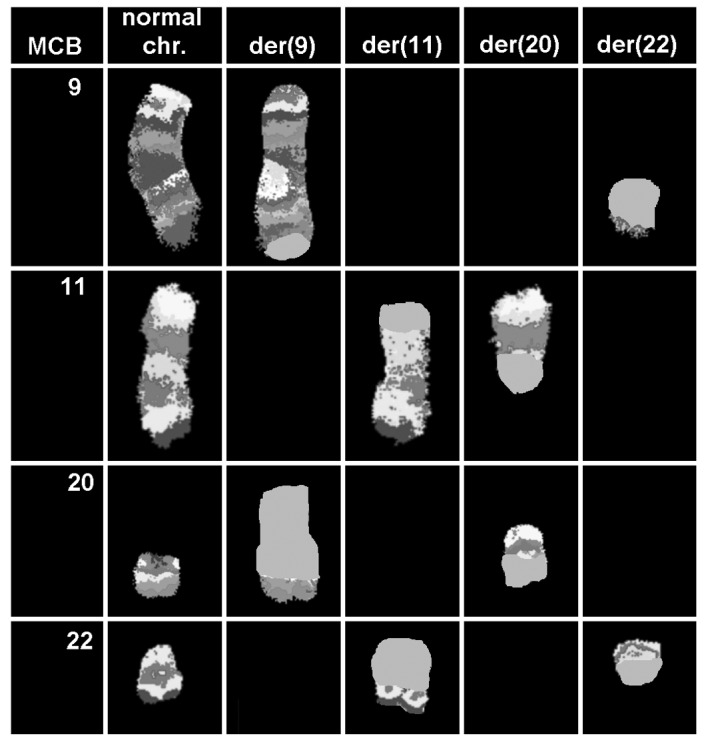
Array-proven multicolor banding (aMCB) was used to determine which chromosomes were involved in the complex rearrangement. Each lane shows the results of aMCB analysis using probe sets for chromosomes 9, 11, 20 and 22. The normal chromosomes are shown in the first column and the derivatives of the four chromosomes in the subsequent ones. The unstained regions on the derivative chromosomes are shown in gray. der, derivative chromosome.

Karyotyping was performed following the chemotherapy treatment. A complex karyotype 46,XX,t(9;11;20;22) was determined by GTG-banding (Fig. 1) and further specified by molecular cytogenetic studies (Figs. 2 and 3). Dual-color FISH using a probe specific for BCR and ABL revealed typical Ph status and the BCR/ABL fusion gene on der(22), while the BCR/ABL fusion gene on der(9) was not observed (Fig. 2A). Dual-color FISH using WCP-specific probes was performed to confirm the rearrangement (Fig. 2B–D). Thus, chromosomes 9, 11, 20 and 22 were found to be involved in the karyotypic changes. aMCB using probes for the corresponding chromosomes was applied as previously reported (13). The presence of a complex translocation among the four chromosomes was confirmed (Fig. 3), and the final karyotype obtained was determined as 46,XX,t(9;11;20;22)(q34;p11.2;q11.21;q11)[20].

RT-PCR analysis of the fusion transcript revealed a band corresponding to the b2a2 transcript (data not shown).

## Discussion

We described a novel Ph-positive CML case with a new complex variant translocation t(9;11;20;22)(q34;p11.2;q11.21;q11)[20]. To the best of our knowledge, this translocation has not been observed in CML previously (15).

In 2–10% of Ph chromosome CML cases, complex trans-locations have been reported in addition to those involving chromosomes 9 and 22 (1). At present, it appears that in such rearrangements any other chromosome may be involved. However, it has been suggested that the distribution of chromosomes and breakpoints is non-random with the chromosomal bands most susceptible to breakage being 1p36, 3p21, 5q31, 6p21, 9q22, 10q22, 11q13, 12p13, 17p13, 17q21, 17q25, 19q13, 21q22, 22q12 and 22q13 (1). The breakpoints 11p11.2 and 20q11.21 have not yet been reported in variant Ph rearrangement (1).

A possible candidate in 11p11.2 is the cluster of differentiation 82 (KAI1/CD82), a human protein encoded by the CD82 gene, originally identified as a putative metastasis suppressor gene (16). KAI1/CD82 is widely expressed in human tissues, and downregulation of this gene is associated with the metastatic phenotype of several malignancies, including carcinomas of the prostate, lung, colon, pancreas, stomach, liver and bladder. Downregulation of KAI1/CD82 is associated with the acquisition of high metastatic properties in Dunning rat prostate cancers (17). The transfer of the KAI1/CD82 gene into mammary cancer cells suppresses their metastatic potential but does not affect primary tumor growth (18).

The breakpoint 20q11.21 was again reported in patients with B-cell precursor acute lymphoblastic leukemia (BCP-ALL) (19,20). The KIF3B gene mapped at position 20q11.21 (19) may be involved in this translocation. It encodes kinesin-like protein KIF3B in humans. KIF3A and KIF3B form a heterodimer that functions as a microtubule-based fast anterograde translocator of membranous organelles (21). The head domain of KIF3A/B, containing the ATPase activity, binds to a microtubule and the tail domain binds to KAP3 (21,22).

Further studies are required to establish which genes, if any, are involved in complex Ph translocations. Notably, in the present case, Ph is associated with a deletion in the derivative chromosome 9. In 2000, Sinclair *et al*(2) reported deletions on der(9)t(9;22) including 3′ BCR and 5′ ABL1 in 10–15% of CML patients and suggested that these findings had adverse prognostic significance. Certain investigators have suggested an association between ASS deletion and resistance to therapy (23). Kreil *et al*(24) demonstrated that derivative chromosome 9 deletions had a heterogeneous prognostic effect on prognosis. Only deletions spanning the ABL1/BCR breakpoint were associated with an adverse prognosis. In this study, we only identified the deletion of ABL1/BCR, and not that of the ASS gene.

In conclusion, we report a novel case of a Ph-positive CML in the chronic phase with an absence of the ABL/BCR fusion gene on der(9) and a new complex variant Ph translocation involving the four chromosomal regions 9q34, 11p11.2, 20q11.21 and 22q11. Notably, the patient concerned showed a good response to imatinib despite loss during follow-up.
